# Exploitation of Filamentous and Picoplanktonic Cyanobacteria for Cosmetic Applications: Potential to Improve Skin Structure and Preserve Dermal Matrix Components

**DOI:** 10.3390/md18090486

**Published:** 2020-09-22

**Authors:** Janaína Morone, Graciliana Lopes, Marco Preto, Vítor Vasconcelos, Rosário Martins

**Affiliations:** 1 Interdisciplinary Centre of Marine and Environmental Research (CIIMAR/CIMAR), University of Porto, Terminal de Cruzeiros do Porto de Leixões, Av. General Norton de Matos s/n, 4450-208 Matosinhos, Portugal; janabavini@ciimar.up.pt (J.M.); glopes@ciimar.up.pt (G.L.); mpreto@ciimar.up.pt (M.P.); vmvascon@fc.up.pt (V.V.); 2 FCUP, Faculty of Sciences, University of Porto, Rua do Campo Alegre, Edifício FC4, 4169-007 Porto, Portugal; 3 Health and Environment Research Centre, School of Health, Polytechnic Institute of Porto, Rua Dr. António Bernardino de Almeida, 400, 4200-072 Porto, Portugal

**Keywords:** cyanobacteria, carotenoids, phenolic content, anti-aging, hyaluronidase, cosmetics

## Abstract

The use of natural products in skin care formulations gained interest as a concern for modern societies. The undesirable side effects of synthetic compounds, as well as the associated environmental hazards, have driven investigation on photosynthetic organisms as sustainable sources of effective and environmentally friendly ingredients. The use of natural extracts in cosmetics has been highlighted and, along with plants and algae, cyanobacteria have come into focus. Due to their low culture demands, high grow rates and ability to produce a wide variability of bioactive metabolites, cyanobacteria emerged as an economic and sustainable base for the cosmetic industry. In this study, we evaluated the potential of ethanol extracts of picocyanobacteria strains of the genera *Cyanobium* and *Synechocysti*s and filamentous strains of the genera *Nodosilinea*, *Phormidium* and *Tychonema* for skin applications, with focus in the field of anti-aging. The extracts were analyzed for their pigment profile, phenolic content, antioxidant potential, cytotoxicity against keratinocytes (HaCat), fibroblasts (3T3L1), endothelial cells (hCMEC/D3) and capacity to inhibit hyaluronidase (HAase). The total carotenoid content ranged from 118.69 to 383.89 μg g^−1^ of dry biomass, and the total phenolic content from 1.07 to 2.45 mg GAE g^−1^. Identified carotenoids consisted of zeaxanthin, lutein, canthaxanthin, echinenone and β-carotene, with zeaxanthin and lutein being the most representative (49.82 and 79.08 μg g^−1^, respectively). The highest antioxidant potential was found for *Phormidium* sp. LEGE 05292 and *Tychonema* sp. LEGE 07196 for superoxide anion radical (O_2_^•−^) scavenging (IC_50_ of 822.70 and 924 μg mL^−1^, respectively). Low or no cytotoxicity were registered. Regarding HAase inhibition, *Tychonema* sp. LEGE 07196 and *Cyanobium* sp. LEGE 07175 showed the best IC_50_ (182.74 and 208.36 μg mL^−1^, respectively). In addition, an increase in fibroblast proliferation was registered with these same strains. From this work, the ethanol extracts of the species *Tychonema* sp. and *Cyanobium* sp. are particularly interesting for their potential application in anti-aging formulations, once they stimulated fibroblast proliferation and inhibit hyaluronic acid digestion.

## 1. Introduction

Cosmetics are part of the daily lives of millions of people. While still linked to aesthetic appearance, the concern over skin health has undergone a considerable increase, resulting in a significant demand for skin care products. The ethical issues associated to animal-derived products and the use of synthetic compounds inducing toxic side effects in humans, such as allergic reactions [1] and environmental hazards [2], have driven the research on cosmetic compounds from photosynthetic organisms [3].

Along with plants [4] and algae [5], cyanobacteria have also come into focus as a source of compounds for skin care applications [3,6]. Through a long evolutionary history based on adaptations to inhospitable environmental conditions, cyanobacteria evolved remarkable secondary metabolism pathways, being among the most prolific microorganisms producing bioactive compounds [7]. While cyanobacteria-based research has been mainly directed to pharmacological and nutraceutical applications, in the last few years, the interest of these organisms in terms of skin protection has increased. In fact, UV protection, moisturizing and antioxidant activity have already been described for cyanobacteria-derived compounds, such as the mycosporine-like amino acids (MAAs) and scytonemin (SCY), exopolysaccharides (EPS) [8] and carotenoids and phenols, respectively. Among them, carotenoids are particularly interesting, once they have demonstrated that they bear potential for major signals of unhealthy and aged skin, presenting anti-inflammatory activity, through the downregulation of the expression of the inducible nitric oxide synthase and of cyclooxygenase 2, also reducing the release of tumor necrosis factor-α (TNF-α), interleukin (IL)-1β and IL-6 [9,10]. Carotenoids are also well recognized for their antioxidant activity, which is a real asset in the skincare framework. Through radical scavenging, carotenoids can reduce the formation of wrinkles, thus reducing tissue damage and accelerating their repair [9].

The process of skin aging is one of the biggest aesthetic concerns especially for those who have mature skin. In the dermis, fibroblasts are responsible for the production of the extracellular matrix, which mainly comprises hyaluronic acid (HA), collagen and elastin, that provide firmness and elasticity to the skin [6]. HA is an important component to promote normal fibroblast activity, being essential to restore the extracellular matrix and maintain skin filling. This glycosaminoglycan keeps collagen fibers alive and allows up-regulation of elastin [11]. In this way, HA improves skin moisture, exerts skin barrier repair activity and provides a dermal filling effect [12]. As we get older, the HA production gradually decreases due to a complex and continuous biological process that is characterized by cellular and molecular changes, and also by extrinsic factors, such as exposure to UV radiation, air pollution, smoke and weather conditions, like wind and cold [6]. In this sense, cosmetic approaches to recover skin filling consists in HA injections, or in the delay of HA degradation through the inhibition of hyaluronidase (HAase).

In the cosmetic industry, some cyanobacteria active extracts are already commercially available with the purpose of providing skin protection such as the Spirulin extract Spiralin ^®^ (Patents nº EP 2 563 478 B1 + US 2014/0127336 A1) used in products such as Skinicer ^®^ Repair cream and Spirularin ^®^ with regenerative effects on damaged skin cells and collagen, and protection against UV radiation. Moreover, from *Aphanizomenon flos-aquae*, the natural colorant effect of the phycobiliprotein C-Phycocyanin (C-PC) has been used in NaturCyanin Bioactive ^®^ (CAS nº 11016-15-2), to replace current synthetic pigments, due to its attractive pink-purple color in final products.

With the exception of one-off studies, the research devoted to the identification and quantification of carotenoids and phenols in cyanobacteria is still scarce, namely regarding picocyanobacteria, such as the genera *Cyanobium* and *Synechocystis* [7]. Moreover, with the exception of *Spirulina*, the exploitation of other cyanobacteria genera for cosmetic attributes is scarce and worth researching. With this aim, a set of cyanobacteria strains isolated from Portuguese environments, and maintained in the Blue Biotechnology and Ecotoxicology culture collection (LEGE CC, http://lege.ciimar.up.pt/) was studied for their potential use in skin care products. A phytochemical and biological analysis of 70% ethanol extracts (compatible with the incorporation in cosmetic formulations) was undertaken to unravel the carotenoid profile, for the quantification of total phenols and for the evaluation of the antioxidant potential. The cytotoxicity of the extracts was evaluated against keratinocytes, the fundamental cells of epidermis; fibroblasts, the primordial cells of the connective tissue of the dermis; and endothelial cells, due to their importance in skin irrigation. The non-toxic extracts, and those with the capacity to stimulate fibroblast proliferation, and consequently dermal matrix regeneration, were selected for further analysis, regarding their capacity to slow down HA degradation through HAase inhibition.

## 2. Results

### 2.1. Phytochemical Analysis

#### 2.1.1. Carotenoids

**Figure 1:**
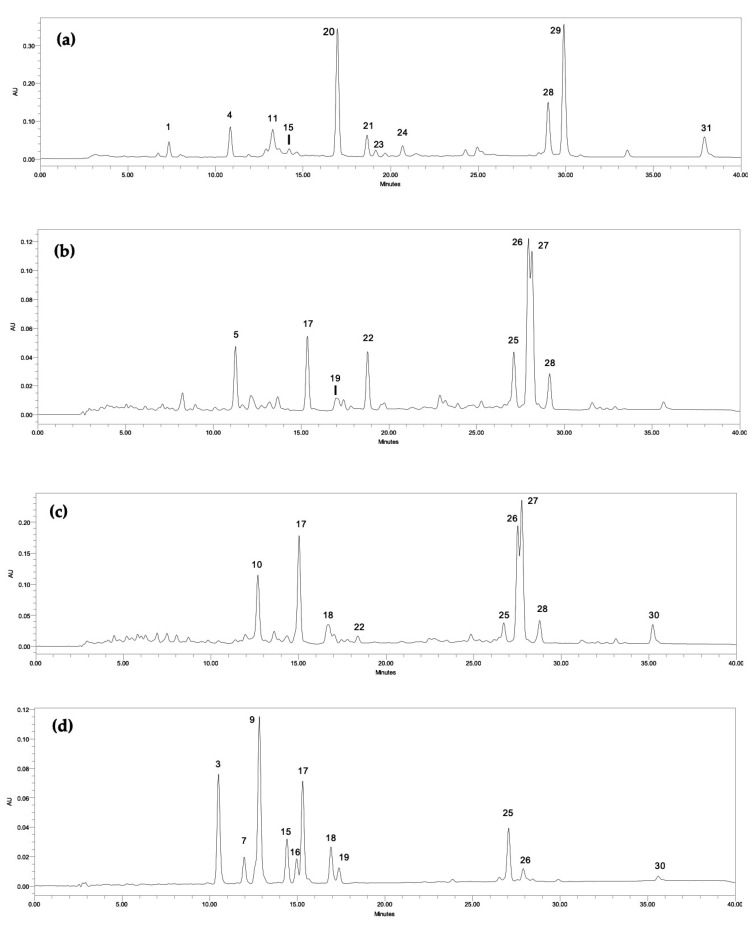
Carotenoid and chlorophyll chromatographic profiles of cyanobacteria ethanol (70%) extracts of (**a**) *Phormidium* sp. LEGE 05292, (**b**) *Tychonema* sp. LEGE 07196, (**c**) *Synechocystis salina* LEGE 06155 and (**d**) *Cyanobium* sp. LEGE 07175, obtained by High Performance Liquid Chromatography with Photo-Diode Array detection (HPLC-PDA) and recorded at 450 nm; (1) unidentified chlorophyll; (3, 9, 20) β-carotene oxygenated derivatives; (4, 5, 10, 11, 24) unidentified carotenoids; (7, 15, 16, 19, 21, 23) lutein derivatives; (17) zeaxanthin; (18) lutein; (22) canthaxanthin; (25 and 27) chlorophyll-*a* derivatives; (26) echinenone; (28) chlorophyll-*a*; (29) echinenone derivative, (30) β-carotene; and (31) β-carotene derivative.

The analysis of the ethanolic extracts from the different cyanobacteria strains by HPLC-PDA allowed the determination of 27 carotenoids, three chlorophyll derivatives and chlorophyll*-a* (Table 1). Figure 1 illustrates the chromatographic profile of four different cyanobacteria genera evaluated in this survey: *Phormidium* sp. LEGE 05292 (a), *Tychonema* sp. LEGE 07196 (b), *Synechocystis salina* LEGE 06155 (c) and *Cyanobium* sp. LEGE 07175 (d). The identified compounds consisted of four xanthophylls (zeaxanthin (17), lutein (18), canthaxanthin (22) and echinenone (26)) and one carotene (β-carotene (30)) as well as chlorophyll-*a* (28). Eleven compounds with the same spectra of the identified carotenoids, but with a different retention time as those of the standard, were also detected in some samples, being tentatively identified as derivatives (3, 7, 9,13,15,16, 19–21, 23 and 29). The same was done for chlorophyll*-a* derivatives (25 and 27) (Table 1). Ten peaks displaying a UV spectrum with different maxima to those of the available standards, but characteristic of carotenoids, were also detected and labelled as unidentified carotenoids (2, 4–6, 8, 10–f12, 14 and 24). One unidentified chlorophyll (1) was detected in *Phormidium* sp. LEGE 05292, whose absorption spectrum is similar to chlorophyll-*c*, as previously identified by other authors [13].

The total carotenoid concentration ranged from 118.69 to 383.89 μg g^−1^ of dry biomass. The highest carotenoid content was found in *S. salina* LEGE 06099 followed by *N. nodulosa* LEGE 06102 and *Phormidium* sp. LEGE 05292 (383.89, 371.43 and 215.88 μg g^−1^, respectively) (*p* < 0.05). Zeaxanthin (17) was found in all samples, with the exception of *Phormidium sp.* LEGE 05292, where only an unidentified β-carotene oxygenated derivative was found (20) (Figure 1a). The highest content of zeaxanthin was registered in the strain *S. salina* LEGE 06099 followed by *N. nodulosa* LEGE 06102 and *Cyanobium* sp. LEGE 06113 (49.82, 39.41 and 25.93 μg g^−1^, respectively).

Lutein (18) was identified in the majority of the analyzed strains, with the exception of *Phormidium* sp. LEGE 05292 and *Tychonema* sp. LEGE 07196, where only lutein derivatives were found (15, 19, 21 and 23) (Figure 1a,b). In the same way as zeaxanthin, the highest lutein content was detected in *S. salina* LEGE 06099, followed by *N. nodulosa* LEGE 06102 and *Cyanobium* sp. LEGE 06113 (79.08; 50.25 and 23.38 μg g^−1^, respectively) (*p* < 0.05). Regarding the strains *S. salina* LEGE 06155 and *Cyanobium* sp. LEGE 07175, no significant differences were found in terms of lutein content (18.94 and 19.91 μg g^−1^, respectively) (Figure 1c,d). Canthaxanthin (22) was the unique xanthophyll that appeared only in two strains, *S. salina* LEGE 06155 (9.96 μ g g^−1^) (Figure 1c) and *Tychonema* sp. LEGE 07196 (37.30 μg g^−1^) (Figure 1b), the last being significantly richer in this xanthophyll (*p* < 0.05).

Regarding echinenone (26), this carotenoid was found in five strains. In *Phormidium* sp. LEGE 05292, only an echinenone derivative was tentatively identified (29) (Figure 1a) and, in *Cyanobium* sp. LEGE 06113, this compound was not detected. The strain *S. salina* LEGE 06155 presented the highest content of echinenone (76.02 μg g^−1^), followed by *Tychonema* sp. LEGE 07196 (58.25 μg g^−1^) and *S. salina* LEGE 06099 (48.37 μg g^−1^) (*p* < 0.05) (Figure 1b,c).

β-carotene (30) was detected in *N. nodulosa* LEGE 06102, with the highest content of 40.76 μg g^−1^ (*p* < 0.05), followed by *S. salina* LEGE 06155 (22.96 μg g^−1^). In *Phormidium* sp. LEGE 05292, in addition to a β-carotene oxygenated derivative (20), another derivative was found (31) (Figure 1a).

Amongst the pigments analyzed, total chlorophylls content, including chlorophyll-*a* (28), their derivatives (25 and 27) and the unidentified chlorophyll (1) were superior to those of carotenoids. The chlorophylls content ranged from 634.71 up to 4762.90 μg g^−1^, the species with the highest amount being *S. salina* LEGE 06155, followed by *Tychonema* sp. LEGE 07196 (3025.52 μg g^−1^) and *Phormidium* sp. LEGE 05292 (2125.04 μg g^−1^) (*p* < 0.05). Regarding chlorophyll-*a*, its highest content was found in the strain *Phormidium* sp. LEGE 05292 (1741.99 μg g^−1^), followed by *S. salina* LEGE 06155 (616.85 μg g^−1^) and *Tychonema* sp. LEGE 07196 (456.56 μg g^−1^) (*p* < 0.05). Besides chlorophyll-*a*, its derivatives were also found in considerably high amounts.

#### 2.1.2. Total Phenolic Content

The total phenolic content (TPC) of the cyanobacteria extracts under study was determined by the colorimetric method of Folin-Ciocalteu and expressed in mg GAE g^−1^ of dry biomass (Table 2). The highest phenolic content was found in *S. salina* LEGE 06099 (2.45 mg GAE g^−1^) (*p* < 0.05), followed by *Phormidium* sp. LEGE 05292 (1.52 mg GAE g^−1^) and *Cyanobium* sp. LEGE 06113 (1.41 mg GAE g^−1^). In the other cyanobacteria strains, the values ranged from 1.07 to 1.23 mg GAE g^−1^, with *Tychonema* sp. LEGE 07196 being the strain with the lowest TPC.

In this work, even though *Synechocystis* sp. LEGE 06155 and *Synechocystis* sp. LEGE 06099 belong to the same genus and were subjected to similar culture conditions, the TPC of the first one was significantly lower than those of the last (1.18 mg GAE g^−1^ and 2.45 mg GAE g^−1^, respectively) (Table 2) as well as for zeaxanthin, lutein and total carotenoid content (*p* < 0.05) (Table 1). Likewise, this was observed for the genus *Cyanobium*, represented by the strains *Cyanobium* sp. LEGE 07175 (1.09 mg GAE mL^−1^) and *Cyanobium* sp. LEGE 06113 (1.41 mg GAE mL^−1^), also significantly different (*p* < 0.05) regarding TPC.

### 2.2. Antioxidant Activity

#### 2.2.1. DPPH^•^ (2,2-diphenyl-1-picrylhydrazyl) Scavenging Activity

**Figure 2:**
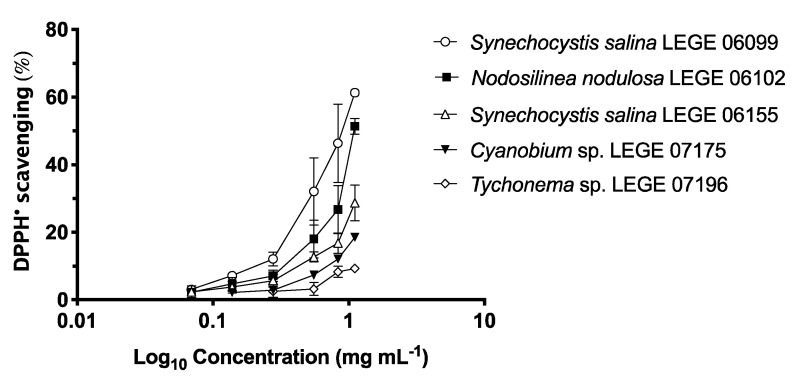
DPPH^•^ scavenging activity of ethanol (70%) extracts of *Synechocystis salina* LEGE 06099, *Nodosilinea nodulosa* LEGE 06102, *Synechocystis salina* LEGE 06155, *Cyanobium* sp. LEGE 07175 and *Tychonema* sp. LEGE 07196. Values are expressed as mean ± SD, *n* = 3.

The results of DPPH^•^ scavenging activity (%) of cyanobacteria ethanolic extracts are presented in Figure 2, and the respective IC values are shown in Table 3. The highest DPPH^•^ scavenging activity was observed with *S. salina* LEGE 06099 (IC_50_ = 863.82 μg mL^−1^), followed by *N. nodulosa* LEGE 06102 (IC_50_ = 1077.59 μg mL^−1^). *S. salina* LEGE 06099 was the strain that showed the highest value for TPC (2.45 mg GAE g^−1^). Similarly, *S. salina* LEGE 06099 was the strain richest in total carotenoids, zeaxanthin and lutein, followed by *N. nodulosa* LEGE 06102. According to the statistical analyses, a significant negative correlation (−0.959, *p* < 0.05) was found between the TPC and the IC_50_ regarding DPPH^•^ scavenging.

#### 2.2.2. O_2_^•−^ Scavenging Activity

**Figure 3:**
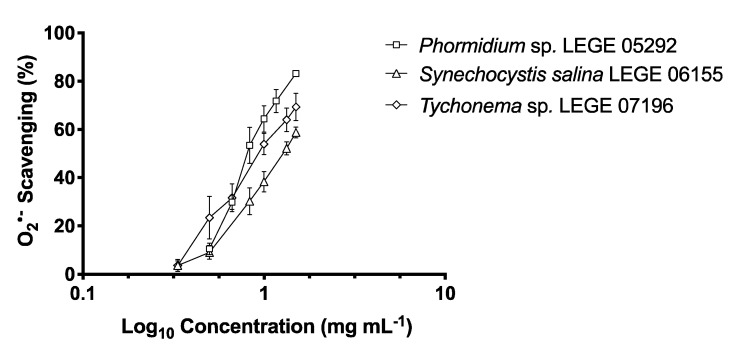
Superoxide radical (O_2_^•−^) scavenging activity of ethanol (70%) extracts of *Phormidium* sp. LEGE 05292, *Synechocystis salina* LEGE 06155 and *Tychonema* sp. LEGE 07196. Values are expressed as mean ± SD, *n* = 3.

Data concerning O_2_^•−^ scavenging capacity are summarized in Table 3 and Figure 3. *Phormidium* sp. LEGE 05292 attained the lowest IC_50_ (822.70 μg mL^−1^) followed by *Tychonema* sp. LEGE 07196 (IC_50_ = 924.21 μg mL^−1^) and *S. salina* LEGE 06155 (IC_50_ = 1275.86 μg mL^−1^). Furthermore, a significant negative correlation between the IC_50_ values and lutein (−1.000, *p* < 0.05), canthaxanthin (−0.954, *p* < 0.01) and β-carotene (−0.955, *p* < 0.01) content was found, although, with no statistical significance regarding total carotenoids, a negative correlation was also noted.

### 2.3. Cytotoxicity Assay

**Figure 4:**
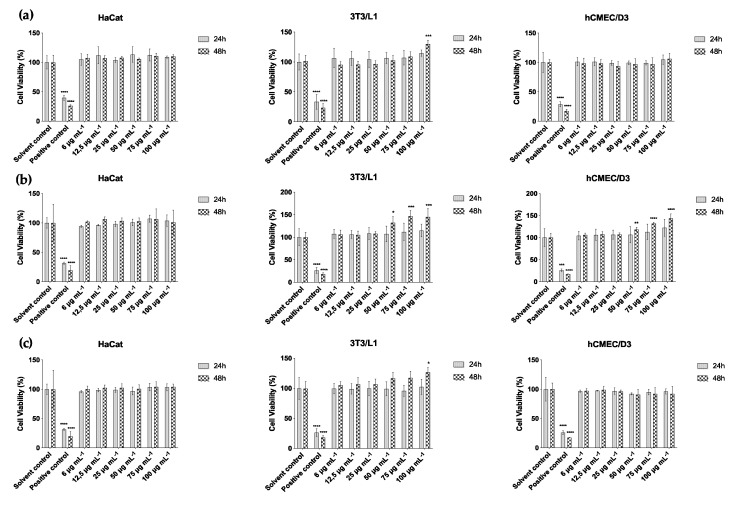
Cell viability after 24 and 48 h of exposure to cyanobacteria ethanol (70%) extracts of (**a**) *Cyanobium* sp. LEGE 07175, (**b**) *Synechocystis salina* LEGE 06155 and (**c**) *Tychonema* sp. LEGE 07196 in the cell lines HaCat, 3T3L1 and hCMEC/D3. Results are expressed as mean ± SD, *n* = 3. Statistical differences at * *p* < 0.05, ** *p* < 0.01, *** *p* < 0.001, **** *p* < 0.0001 (One way ANOVA).

Considering the cellular assays, cytotoxic effects were only registered for the strain *Phormidium* sp. LEGE 05292 at 100 μg mL^−1^ in keratinocytes and, at 100 and 75 μg mL^−1^ in fibroblasts, with no cytotoxic effect on the endothelial cells. Nevertheless, with some of the other strains and cell lines, an increase in cell viability occurred (Figure 4). The highest percentage of cell viability was observed in fibroblasts exposed to the ethanol extract of the strain *S. salina* LEGE 06155 (*p* < 0.001) after 48 h of exposure, with a percentage of cell viability of about 145% for extract concentrations of 100 and 75 μg (dry extract) mL^−1^, and 132% for an extract concentration of 50 μg mL^−1^ (*p* < 0.05), and then followed up with the average of 100% in the lower concentrations (Figure 4b). A significant increase in fibroblast viability was also observed for the strain *Cyanobium* sp. LEGE 07175 and for *Tychonema* sp. LEGE 07196, with 130% (*p* < 0.001) and 127% (*p* < 0.05) of cell viability for an extract concentration of 100 μg mL^−1^, respectively (Figure 4a). Regarding endothelial cells, the strain *S. salina* LEGE 06155 was the only one that showed a high percentage of cell viability for concentrations of 100, 75 and 50 μg mL^−1^, after 48 h (144, 132 and 118%, respectively) (Figure 4b).

### 2.4. Hyaluronidase Inhibition

Under oxidative stress, HAase, the enzyme responsible for the depolymerisation of HA, is over-activated and excessively breaks down HA, which is a polysaccharide present in the extracellular matrix of the connective tissue and has an important role in the propagation and exacerbation of allergic, inflammatory and infectious states [14]. Thus, HAase inhibitors can combine anti-allergic, anticancer and anti-aging activities, contributing to maintain the normal skin structure, as well as its barrier function. After cytotoxicity assays, the strains that demonstrated potential to stimulate fibroblast proliferation, e.g., *Cyanobium* sp. LEGE 07175 and *Tychonema* sp. LEGE 07196, were chosen to perform the HAase inhibition assay. While *Synechocistis salina* LEGE 06155 extract also stimulated fibroblast proliferation, it was disregarded from the following studies because of its capacity to stimulate endothelial cell overgrowth and possible negative interference in tumor environments [15].

**Figure 5:**
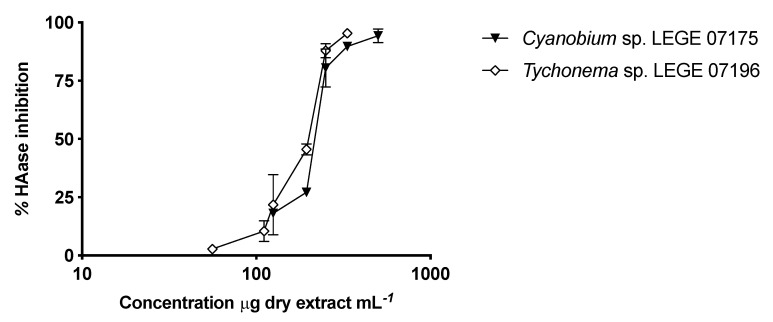
Inhibitory activity of cyanobacteria ethanol (70%) extracts of *Cyanobium* sp. LEGE 07175 and *Tychonema* sp. LEGE 07196 against hyaluronidase (HAase). Results are expressed as percentage of HAase inhibition relative to control. Values are expressed as mean ± SD, *n* = 3.

Regarding HAase inhibition, both species studied herein, *Cyanobium* sp. LEGE 07175 and *Tychonema* sp. LEGE 07196, showed high potential. Even though the highest inhibitory activity was found for *Tychonema* sp. LEGE 07196 (IC_50_ = 182.74 ± 21.17μg mL^−1^), *Cyanobium* sp. LEGE 07175 also presented a good IC_50_ of 208.36 ± 22.06 μg mL^−1^ (Figure 5).

## 3. Discussion

Carotenoids and phenolic compounds are phytochemicals with a wide range of chemical and biological functions, namely the capability of scavenging highly reactive free radicals, acting as natural antioxidants through a non-enzymatic mechanism [16]. In cyanobacteria, these compounds appear to play important roles in the adaptive response to oxidative stress resulting from constant exposure to UV radiation [17]. Due to their slow growth in environmental conditions, the picoplanktonic *Cyanobium* and *Synechocystis* and the filamentous *Nodosilinea*, *Phormidium* and *Tychonema* have been largely overlooked. These genera of cyanobacteria represent a large fraction of the marine cyanobacterial strains isolated from the Portuguese coasts, which are worth of further exploitation. Considering the results obtained herein, it is obvious that the carotenoid composition varied between the strains. Zeaxanthin was found in all strains, except in *Phormidium* sp. LEGE 05292, and lutein or lutein derivatives were found in all strains. Both carotenoids were found in higher concentrations than those previously reported for other cyanobacteria strains. For zeaxanthin, contents obtained in this study ranged from 49.82 to 7.97 μg g^−1^, whereas cyanobacteria such as *Anabaena vaginicola* and *Nostoc*, values from 46.4 μg g^−1^ to 15.5 μg g^−1^, respectively, were described [18]. For lutein, the content found in the cyanobacteria analyzed in the present study ranged between 79.08 and 18.94 μg g^−1^, while contents of 7.7 and 0.8 μg g^−1^ were described for *Anabaena* sp. and *Nostoc* sp., respectively, [18] and 0.822 μg g^−1^ in *Gloeothece* sp. [19]. Zeaxanthin is a xanthophyll which has a photoprotective role in cyanobacteria, which justifies its broad occurrence among different cyanobacteria genera. In humans, zeaxanthin was found to reduce oxidative damage in the eyes and to prevent age-related macular degeneration [20]. Lutein is also largely found in cyanobacteria. This xanthophyll was also found to decrease the generation of reactive oxygen species (ROS) in the skin, thus protecting the epidermis and dermal layers against the deleterious effects of UVs [21], besides being also involved in eye protection by preventing cataracts [22].

Echinenone or its derivatives were also found in the majority of the cyanobacteria strains analyzed. Echinenone is described as an essential carotenoid involved in photoprotection due to a carbonyl group, which seems to be substantially essential for photoprotection [23]. Kusama et al. [24], using mutants of *Synechocystis* sp. deficient in echinenone and zeaxanthin, registered a stimulation in the production of singlet oxygen and therefore concluded that these carotenoids might be involved in the protection of PSII against photoinhibition. In the present study, the highest content of echinenone (76.02 μg g^−1^) was detected in strain *S. salina* LEGE 06155, followed by *Tychonema* sp. LEGE 07196 (58.25 μg g^−1^). Echinenone isomers were identified in *Aphanothece* sp. in higher amounts than that reported in this study (396.44 and 200.10 μg g^−1^ for all-trans-echinenone and 9-cis-echinenone, respectively) [25]. While this compound was not found in the ethanolic extract of *Cyanobium* sp. LEGE 06113, Pagels et al., 2020 [26], reported that acetonic extract of *Cyanobium* sp. LEGE 06113 is rich in echinenone (1.12 mg g^−1^). Furthermore, Lopes et al., 2020 [10], showed that acetonic extracts of this genus are also rich in echinenone. The authors proved the effectiveness of this solvent in the extraction of carotenoids. In addition, another study [27] showed that, when analyzing acetonic and aqueous extracts of *Cyanobium* sp., a positive correlation between pigment profiles and antioxidant capacity was found.

Regarding canthaxanthin, this carotenoid was only found in *S. salina* LEGE 06155 and *Tychonema* sp. LEGE 07196, the last being significantly richer in this xanthophyll (9.96 and 37.30 μg g^−1^, respectively). A reference to this same xanthophyll in cyanobacteria was only found for *Aphanothece* sp., with a 51.57 μg g^−1^ content [25]. The results obtained seem to be interesting since this carotenoid was found to suppress skin papilloma [28], besides inducing apoptosis in cancer cell lines [29], which highlights its potential use in humans health.

Another important terpene detected was β-carotene, which was also reported for other cyanobacteria genera such as *Gloeothece* [19], *Aphanothece* [25], *Anabaena* and *Nostoc* [18]. In this work, β-carotene was also found in most of the strains. While in very different concentrations among strains, the production of this compound is an added value, due to its relevance as an anticancer, antidiabetic and antioxidant agent [9]. To our knowledge, there is a lack of previous reports focused to the carotenoid profile of the cyanobacteria genera analyzed herein. Recently, Pagels et al., 2020 [26], reported that acetone and acetone after aqueous extraction from *Cyanobium* sp. LEGE 06113 are rich not only in β-carotene (1.40 and 1.46 mg g^−1^), but also in pigments such as echinenone, lutein and zeaxanthin. Therefore, our work may corroborate that cyanobacteria of the genera *Cyanobium, Synechocystis, Nodosilinea, Phormidium* and *Tychonema* are rich in carotenoids, which puts some light on the potential of these species to be used in skin care formulations.

In addition to carotenoids, several studies have reported the beneficial effects of chlorophylls, namely as antioxidant agents [30]. Chlorophylls are one of the useful bioactive compounds extracted from microalgae and widely used in food, cosmetic and pharmaceutical industries [31]. Abundantly present in nature due to the important role in photosynthesis, chlorophylls are also one of the main pigments in cyanobacteria. The results obtained in this study demonstrate that the strains are rich in this pigment in values similar to those found for other cyanobacteria and microalgae [32,33]. The highest content of chlorophylls was detected in *Synechocystis salina* LEGE 06155, followed by *Tychonema* sp. LEGE 07196 (4762.90 and 3025.52 μg g^−1^, respectively).

Studies concerning the phenolic content of cyanobacteria were mainly performed with filamentous freshwater and terrestrial forms, such as the genera *Anabaena*, *Lyngbya*, *Nostoc*, *Oscillatoria* and *Spirulina* [34,35,36,37], which are able to grow in large densities and are exposed to stress conditions, such as desiccation and high temperatures. Besides abiotic factors, other factors can influence species metabolome regarding TPC. For instance, in the present study, *S. salina* LEGE 06099 and *S. salina* LEGE 06155 presented significant differences regarding TPC, even belonging to the same species. This can be explained, at least in part, because *S. salina* LEGE 06099 comes from the intertidal zone, while *S. salina* LEGE 06155 was isolated from a rock surface, where there is greater sun exposure and stressors, favoring the production of such compounds. In addition, even belonging to the same species, the different environments from which these species belong result in differences in their genetic codes (represented by the different LEGE codes), which may influence their metabolome. Considering strains of the genera *Cyanobium*, *Nodosilinea* and *Tychonema*, to the best of our knowledge, there are no previous reports regarding the TPC content. Comparing the present results with other TPC results, most have only reported slightly higher phenolic content, such as for *Oscillatoria* sp. strains (3.9 to 5.0 mg GAE g^−1^), *Phormidium* sp. (8.5 mg GAE g^−1^) [36] and cell-free extracts of *Phormidium* sp. (5.43 mg GAE g^−1^) [38].

A previous study [26] with acetonic and aqueous extracts of *Cyanobium* sp. LEGE 06113 also showed promising results, with values around 83 mg GAE g^−1^ of dry extract. In this sense, studies with the genus *Cyanobium* for the cosmetic industry field have proved to be of considerable importance.

One of the main properties of photosynthetic organism to human needs is based on the antioxidant potential of their extracts, since living cells can generate free radicals as products of physiological and biochemical processes. In this work the antioxidant potential of the cyanobacteria strains was determined by the DPPH^•^ scavenging assay and the O_2_^•−^ scavenging assay. The DPPH^•^ assay is based on the reduction of DPPH^•^, by its ability to accept an electron or an azote atom and thus, become a non-radical species, in the presence of molecules with radical scavenging activity. This simple, sensitive and economic method has been widely used to evaluate free radical scavenging activity of phenolic compounds and consequently, to determine the potential antioxidant activity of phenol-containing extracts [39]. Another important free radical is O_2_^•−^, which represents one of the major ROS causing oxidative damage to the human body. In vitro, O_2_^•−^ can be generated by the NADH/ PMS system and measured spectrophotometrically [40].

The IC_50_ values regarding DPPH^•^ scavenging capacity were not as low as those reported in literature for other cyanobacteria genera. A previous study [41] reported that methanolic extracts of three *Synechocystis* sp. strains and one *Oscillatoria* sp. exhibited IC_50_ values of 54.59; 56.79; 65.16 and 78.43 μg mL^−1^, respectively. Another study [36] reported antioxidant values of 83.08 μg mL^−1^ for phenolic extracts of *Phormidium* sp. and of 92.23 μg mL^−1^ for *Oscillatoria* sp. Despite species-specific factors, these results can be explained, at least in part, by the solvents used in extracts preparation, since different polarities lead to the extraction of different compounds, and thus to a different matrix effect that can affect the bioavailability of the phytochemicals [42]. Moreover, total chlorophylls, for example, were found to interfere with the determination of radical scavenging capacity [19]. Thus, the results obtained for the strain *Phormidium* sp. LEGE 05292 might be due to its chlorophyll content, being this the richest strain in total chlorophylls. Nevertheless, a significant negative correlation between the IC values for DPPH^•^ scavenging and the total carotenoids content was observed (−0.824, *p* < 0.01). Weak negative correlations between the antioxidant capacity and TPC were also reported in previous studies [35]. Consequently, it can be inferred that crude extracts might not only provide carotenoids and phenols, but a complex mixture of compounds that may also have antioxidant potential, such as fatty acids, exopolymers and phycocyanin [43,44]. The results obtained herein with the strain *Phormidium* sp. LEGE 05292 also corroborate these finding, since DPPH^•^ scavenging capacity was not registered despite of a TPC of 1.52 mg GAE g^−1^. For the same strain a controversial result was observed, since the ethanol extract of this species presented the lowest IC_50_ value for O_2_^•^^−^ scavenge and no capacity to scavenge DPPH^•^. This behavior can be tentatively justified by the interference that the extract color exerts in the DPPH^•^ assay, or by a higher capacity of the extract to scavenge oxygen radicals in detriment of nitrogen radicals. Nevertheless, a significant negative correlation between O_2_^•^^−^ IC_50_ values and lutein (−1.000, *p* < 0.05), canthaxanthin (−0.954, *p* < 0.01) and β-carotene (−0.955, *p* < 0.01) content was found.

Cytotoxicity assays were performed using keratinocytes, fibroblasts and endothelial cells. Epidermis, the superficial layer of the skin and responsible for the primary defense against UV radiation, consists in several layers of keratinocytes. To protect against exogenous factors, the skin promotes physiological regenerative events including the proliferation of keratinocytes, leading to epidermal thickening [45]. As previously mentioned, fibroblasts produce important skin components, such as collagen, elastin and HA, providing firmness, regeneration and moisturizing to the skin, being thus targets of cosmetic compounds [46]. The proliferation of fibroblasts, and the increase of the antioxidant potential, are key mechanisms to delay skin aging [47]. The skin has an extensive vascular network which regulates the blood supply for its normal physiology and regeneration, being its deregulation the basis of several human diseases [48]. Overall, the increase in cell viability observed in fibroblasts exposed to *Tychonema* sp. LEGE 07196 and also *Cyanobium* sp. LEGE 07175 reveals that these strains appear to provide stimulation of fibroblast growth and, consequently, the production of the matrix fibers such as collagen, which highlight the potential of the strains in skin care. Furthermore, both species showed capacity for HAase inhibition, making them interesting in combating skin aging, through a potential to preserve dermal matrix components.

Considering all the results obtained in this study, *Tychonema* sp. LEGE 07196 and *Cyanobium* sp. LEGE 07175 showed strong inhibitory activity against HAase (IC_50_ of 182.74 and 208.36 μg mL^−1^, respectively) when compared to the reference drug Di-sodium cromoglicate (DSCG), IC_50_ = 1.105 mg mL^−1^, as previously reported by us [14] in addition to significantly increasing the fibroblasts viability at 100 μg mL^−1^. These results can be compared with a study [49] that reported HAase inhibition (IC_50_ values of 0.15 mg mL^−1^) for an ethanol-insoluble fraction of *Spirulina platensis.* Another study [50] reported that a polysaccharide of the edible cyanobacteria *Nostochopsis lobatus* showed a high HAase inhibitory effect. Thus, this supports the use of cyanobacteria extracts as anti-aging ingredients in the cosmetic industry.

The current research infers that cyanobacteria strains, namely *Cyanobium* sp. LEGE 07175 and *Tychonema* sp. LEGE 07196 can be suggested for cosmeceutical application due to their potential to provide antiaging effects. These findings warrant an expansion of cyanobacteria genera in the exploitation of natural sources for skin formulations to delay skin aging. Moreover, Pagels et al., 2020 [27], studying the strain *Cyanobium* sp., showed that the application of two-phase light cultivation systems may promote pigment production. The authors showed that, after optimized cultivation conditions for the strain *Cyanobium* sp., an increase in biomass, total phycobiliproteins and carotenoid production were clearly observed. Thus, cyanobacteria stand out among other natural sources, such as macroalgae and higher plants, for exhibiting higher growth rates [8]; cultivation requires only basic nutrients and does not depend on arable land [51], which makes the process for obtaining biomass more economical and environmentally sound. 

## 4. Materials and Methods

### 4.1. Cyanobacteria Strains

Seven cyanobacteria strains isolated from Portuguese marine and freshwater ecosystems and maintained in the Blue Biotechnology and Ecotoxicology Culture Collection (LEGECC) at the Interdisciplinary Centre of Marine and Environmental Research (CIIMAR/CIMAR) [52] were chosen taking into account the selection of different genera, and the previous results revealing bioactivities of several cyanobacteria fractions [53], in order to be able to make a more comprehensive screening of the most promising cyanobacteria species. The strains panel included the picoplanktonic *Cyanobium* sp. LEGE 06113, *Cyanobium* sp. LEGE 07175, *Synechocystis salina* LEGE 06099 and *Synechocystis salina* LEGE 06155, and the filamentous *Phormidium* sp. LEGE 05292, *Nodosilinea nodulosa* LEGE 06102 and *Tychonema* sp. LEGE 07196.

### 4.2. Cyanobacteria Biomass: Culture and Harvest

Raw biological material from each cyanobacteria strain was inoculated in liquid Z8 medium, supplemented with 10 μg L^−1^ vitamin B12 and 25 g L^−1^ NaCl for marine strains [54], and cultivated as described by Freitas et al., 2016 [55]. Biomass was concentrated by centrifugation at 7000 Gs for 15 min at 4 °C (Sorvall^TM^ BIOS 16 Bioprocessing Centrifuge, Thermo Fisher Scientific, Bremen, Germany). For marine strains, concentrated biomass was washed with distilled water in order to remove NaCl. The fresh concentrated biomass was frozen, freeze-dried (Telstar LyoQuest) and stored dried at −20 °C. 

### 4.3. Extract Preparation

Cyanobacteria dry biomass was suspended in ethanol (70%) and sonicated at a frequency of 70/80 Hz for 3 min (Vibra-Cell^TM^ ultrasonic liquid processor, Sonics & Materials, INC., Newtown, USA). Cell debris were removed by centrifugation (15,000 Gs, 10 min, 4 °C) (Gyrozen^TM^ 2236R, Vita Scientific, South Korea), and the extraction process repeated five times. Supernatants from each strain were combined and evaporated under reduced pressure (Centrivap Vaccum Benchtop Concentrator, Labconco, KS, USA). The dry extracts were kept at −20 °C until analysis.

### 4.4. Phytochemical Analysis

#### 4.4.1. Determination of Pigments Profile by HPLC-PDA

Dried cyanobacteria extracts were dissolved in methanol (HPLC-grade) to a final concentration of 10 mg mL^−1^ and filtered through a 0.2 μm pore membrane (Pall Corporation, New York, NY, USA). Carotenoid analysis was performed following the method previously described [10,19], with slight modifications. High Performance Liquid Chromatography (HPLC) with photo diode-array (PDA) detector (Waters Alliance 2695 Separations Module, Waters Corporation, Milford, MA, USA) was employed to resolve, detect and identify the compounds of interest. The stationary phase was a Luna 5 μm C18 100A (250–4.6 mm; Phenomenex) column, kept at constant temperature (25 °C) with a column heater (Waters Corporation, Milford, USA). The mobile phase consisted of two solvents: ethyl acetate (A) and acetonitrile:water 9:1 (*v/v*) (B) starting with 100% B and installing a gradient to obtain 40% B at 31 min, 40% B at 36 min, 0% B at 38 min and 0% B from 38 to 55 min. The flow rate was 1 mL min^−1^ and the injection volume was 20 μL. Data were processed using Empower chromatography 2 software (Waters, Milford, USA). Spectra data from all peaks were collected in the range 250 to 750 nm.

Compounds were identified by comparing their retention times and UV-Vis spectra with those of authentic standards. Carotenoid quantification was achieved by measuring the absorbance recorded in the chromatograms relative to external standards at 450 nm. 

Zeaxanthin, lutein, canthaxanthin, echinenone, β-carotene and chlorophyll-a (Extrasynthese, Genay, France and Sigma-Aldrich, St. Louise, MO, USA) were quantified with the authentic standards; unidentified carotenoids were quantified as zeaxanthin, the most common xanthophyll, chlorophyll derivatives as chlorophyll*-a*, the major cyanobacteria chlorophyll, and β-carotene derivatives as β-carotene. Calibration curves were obtained with standard solutions corresponding to five different concentrations, selected as representative of the range of compound concentrations in the samples. The calibration plots, *r^2^* values, the limit of detection (LOD = 3So/b) and the limit of quantification (LOQ = 10So/b, where So is the standard deviation of signal-to-noise ratio and b is the slope of the calibration plot) for the analyzed carotenoids and chlorophyll-*a* are shown in the Table 4.

#### 4.4.2. Determination of Total Phenolic Content (TPC)

The TPC of the cyanobacterial extracts was determined using the colorimetric assay of Folin-Ciocalteu, according to Barroso et al. [56], with some modifications. Briefly, a volume of 25 μL of each extract (10 mg mL^−1^) was thoroughly mixed with 25 μL of Folin-Ciocalteu reagent (Sigma-Aldrich, St. Louis, MO, USA), 200 μL of Na_2_CO_3_ solution (75 g L^−1^) and 500 μL of deionized water. After the incubation period (60 min at room temperature), the absorbance of the colored product was measured at 725 nm, using a Synergy HT Multi-detection microplate reader operated by GEN5^TM^ (Biotek, Bad Friedrichshall, Germany). A standard calibration curve (y = 1.951*x* + 0.01135; R^2^ = 0.9989) was obtained with seven concentrations of gallic acid (GA) (0.025 to 0.5mg mL^−1^). Total phenols in each extract were expressed in mg gallic acid equivalents (GAE) g^−1^ dry biomass. The experiment was carried out in triplicate.

### 4.5. Antioxidant Assays

#### 4.5.1. DPPH^•^ Scavenging Activity

The 2,2-diphenyl-1-picrylhydrazyl (DPPH^•^) scavenging assay was performed as reported before [57], with some modifications. A volume of 25 μL of extract serial dilutions was mixed with 200 μL of 100 μM DPPH^•^ reagent, freshly prepared in methanol [58] in a 96-well plate. GA was used as positive control, and DMSO as negative control. The plate was incubated in the dark, at room temperature, for 15 min. The absorbance of the extracts (dilutions prepared in DMSO) was measured at 515 nm using a Synergy HT Multi-detection Microplate Reader operated with GEN5^TM^ (Biotek, Bad Friedrichshall, Germany). Three independent assays were performed in duplicate. Results were expressed as percentage of radical scavenging face to the untreated control.

#### 4.5.2. Superoxide Anion Radical (O_2_^•−^) Scavenging Activity 

The superoxide anion radical (O_2_^•−^) scavenging activity of the extracts was determined as described previously [59] with some modifications. A volume of 50 µL of serial dilutions of the cyanobacteria extracts (0.33 to 1.66 mg mL^−1^) was mixed with 50 µL of 166 µM β-nicotinamide adenine dinucleotide reduced form (NADH) solution and 150 µL of 43 µM nitrotetrazolium blue chloride (NBT) in a 96 wells plate. A volume of 50 µL of 2.7 µM phenazine methosulphate (PMS) was added to each well, and the radical scavenging activity of the samples monitored with a Synergy HT Multi-detection Microplate Reader operated by GEN5^TM^ (Biotek, Bad Friedrichshall, Germany), in kinetic function, at room temperature, for 2 min, at 562 nm. All reagents were dissolved in phosphate buffer (19 μM, pH 7.4). Three independent assays were performed in triplicate. GA was used as positive control. Results were expressed as percentage of radical scavenging face to the untreated control.

### 4.6. Cell Culture and Cytotoxicity Analysis

#### 4.6.1. Cell Culture

Cytotoxicity assays were performed with the HaCat keratinocyte cell line (ATCC), the 3T3L1 fibroblast cell line (ATCC) and the hCMEC/D3 endothelial cell line (kindly donated by Dr. P. O. Couraud (INSERM, Paris, France)). Cells were cultured in DMEM Glutamax medium (Dulbecco’s Modified Eagle Medium DMEM GlutaMAXTM—Gibco, Massachusetts, USA), supplemented with 10% (*v/v*) fetal bovine serum (Biochrom, Berlin, Germany), 0.1% Amphotericin B (GE Healthcare, Little Chafont, United Kingdom) and 1% Pen–Strep (penicillin–streptomycin, 100 IU m^−1^ and 10 mg mL^−1^, respectively) (Biochrom, Berlin, Germany). Culture and incubation were performed in a 5% CO_2_ humidified atmosphere and at 37 °C.

#### 4.6.2. Cytotoxicity Assay—MTT Assay

The 3-(4,5-dimethylthiazole-2-yl)-2,5-diphenyltetrazolium bromide (MTT) cytotoxicity assay was performed as previously described [55]. In brief, keratinocytes, fibroblasts and endothelial cells were seeded in 96 wells plates at a density of 2.5 × 10^4^ cells mL^−1^, 3.3 × 10^4^ cells mL^−1^ and 1.0 × 10^5^ cells mL^−1^, respectively. After 24 h of adhesion, cells were exposed for 24 and 48 h to fresh medium supplemented with 1% of extract to final concentrations of 6.0 to 100 μg mL^−1^. DMSO at 1% and 20% was tested as solvent (reference for 100% cell viability) and positive (reference for cell death) control, respectively. After each incubation time, 20 μL of 1 mg mL^−1^ MTT (Sigma-Aldrich) was added to each well and incubated for 3 h. Following incubation, the purple colored formazan salts were dissolved in DMSO and the absorbance was read at 550 nm in a Synergy HT Multi-detection microplate reader (Biotek, Bad Friedrichshall, Germany) operated by GEN5^TM^ software. The assay was run in quadruplicate and averaged. Cytotoxicity was expressed as a percentage of cell viability, considering 100% viability in the solvent control. For reproducibility, each assay was independently repeated three times.

### 4.7. HAase Inhibition Assay 

HAase inhibition assay was determined as reported before [14] with some modifications. Twenty-five microliters of serial dilutions of each sample prepared in 70% ethanol were added to each reaction tube. A volume of 175 µL of HA solution (0.7 mg mL^−1^ in water:buffer, 5:2 *v*/*v*, kept at 37 °C) was added to each reaction tube and gently mixed. The reaction was started by adding 25 μL of HAase (900 U/mL in NaCl 0.9%). After 30 min incubation at 37 °C, the enzymatic reaction was stopped with 25 μL of disodium tetraborate 0.8 M, followed by subsequent heating for 3 min in a boiling water bath. After cooling to room temperature, 375 µL of DMBA solution was added and gently mixed (2 g of DMAB dissolved in a mixture of 2.5 mL of 10 N HCl and 17.5 mL of glacial acetic acid and further diluted 1:2 with glacial acetic acid immediately before use). The tubes were incubated at 37 °C for 20 min and the absorbance of the colored product was measured at 560 nm in a Synergy HT Multi-detection microplate reader (Biotek, Bad Friedrichshall, Germany) operated by GEN5^TM^ software. Three independent assays were performed in triplicate. The range of sample concentrations was between 0.056 and 0.5 mg mL^−1^. Di-sodium cromoglicate (DSCG) was used as positive control.

### 4.8. Statistical Analysis

Statistical analysis was performed using IBM SPSS STATISTICS software, version 25.0, IBM Corporation, New York, NY, USA (2011). Data were analyzed for normality and homogeneity of variances by Kolmogorov–Smirnov and Leven’s tests, and then submitted to one-way ANOVA, using a Tukey’s HSD (honest significant difference) as a post hoc test, for total phenols, O_2_^•−^, DPPH^•^ (IC_25_), compounds 3, 7, 9, 13, 15, 17–19, 25, 26, 28 and 30, total carotenoids and total chlorophylls and cell viability, or to a two-tailed unpaired *t*-test to compare the IC_50_ values for DPPH^•^ scavenging assay, and the total amount of compounds 2, 4, 5, 10, 12, 14, 22 and 27. IC_50_ values (expressed in μg of lyophilized extract mL^−1^) were presented as mean ± SD of at least three independent experiments. A Pearson correlation test was used to compare normalized expression data between the chemical profile and the biological activities of cyanobacteria extracts.

## 5. Conclusions

Cyanobacteria are capable of adapting to diverse and extreme environmental conditions, allowing them to produce different secondary metabolites which provide their protection and survival capacity. Besides this, cyanobacteria-derived compounds have also revealed several biological activities with interest for human health and well-being. This study emphasized the capacity of picoplanktonic and filamentous cyanobacteria to produce bioactive compounds with potential use in the cosmetic industry. Overall, cyanobacteria extracts explored in this work demonstrated an ability to scavenge deleterious free radicals implicated in skin aging, to increase fibroblasts viability, foreseeing their potential for dermal matrix fill and consequent wrinkle fading, and also their capacity to delay HA degradation through HAase inhibition. Hereupon, cyanobacteria may be seen as natural and eco-friendly sources of bioactive metabolites with interest for cosmetic industry investment. In addition, cultures can be performed continuously, which is considered the most feasible system for large-scale production, making the process cost effective.

## Figures and Tables

**Table 1:** Carotenoid and chlorophyll contents (μg g^−1^ dry biomass) in the ethanol extracts of the cyanobacteria strains, determined by HPLC-PDA ^1,2,3^.

Peak	Compound	RT (min)	*Phormidium* sp. LEGE 05292	*Synechocystis salina* LEGE 06099	*Nodosilinea nodulosa* LEGE 06102	*Cyanobium* sp. LEGE 06113	*Synechocystis salina* LEGE 06155	*Cyanobium* sp. LEGE 07175	*Tychonema* sp. LEGE 07196
1	Unidentified Chlorophyll	7.36	383.04 ± 44.56	nd	nd	nd	nd	nd	nd
2	Unidentified carotenoid	9.71	nd	nd	21.95 ± 0.17 ^a^	nd	nd	nd	nd
3	β-Carotene oxygenated derivative	10.35	nd	nd	12.58 ± 0.16 ^c^	20.79 ± 0.14 ^a^	nd	16.83 ± ≤ 0.08 ^b^	nd
4	Unidentified carotenoid	10.85	6.73 ± ≤ 0.04 ^b^	13.26 ± 0.42 ^a^	nd	nd	nd	nd	nd
5	Unidentified carotenoid	11.27	nd	nd	nd	8.13 ± ≤ 0.07 ^a^	nd	nd	7.63 ± 0.21 ^b^
6	Unidentified carotenoid	11.59	nd	nd	13.80 ± ≤ 0.02	nd	nd	nd	nd
7	Lutein derivative	12.02	nd	nd	nd	18.02 ± 0.31 ^a^	nd	14.32 ± ≤ 0.07 ^b^	nd
8	Unidentified carotenoid	12.40	nd	nd	63.07 ± 0.23	nd	nd	nd	nd
9	β-Carotene oxygenated derivative	12.51	nd	nd	nd	38.95 ± 0.41 ^a^	nd	25.53 ± 0.11 ^b^	nd
10	Unidentified carotenoid	12.76	nd	118.17 ± 1.04 ^a^	nd	nd	14.62 ± 0.89 ^b^	nd	nd
11	Unidentified carotenoid	13.25	8.20 ± 0.54	nd	nd	nd	nd	nd	nd
12	Unidentified carotenoid	13.67	nd	17.01 ± 0.15 ^a^	12.29 ± 0.14 ^b^	nd	nd	nd	nd
13	Lutein derivative	13.91	nd	nd	43.73 ± 0.84 ^a^	29.59 ± 0.16 ^b^	nd	nd	nd
14	Unidentified carotenoid	14.44	nd	39.18 ± 1.43 ^a^	12.05 ± 0.30 ^b^	nd	nd	nd	nd
15	Lutein derivative	14.72	8.47 ± 0.58 ^b^	nd	nd	nd	nd	21.54 ± 0.29 ^a^	nd
16	Lutein derivative	15.01	nd	nd	nd	nd	nd	12.66 ± 0.65	nd
17	Zeaxanthin	15.36	nd	49.82 ± 1.36 ^a^	39.41 ± 0.06 ^b^	25.93 ± 0.22 ^c^	19.93 ± 0.16 ^d^	16.31 ± 0.23 ^e^	7.97 ± ≤ 0.06 ^f^
18	Lutein	16.33	nd	79.08 ± 0.44 ^a^	50.25 ± 0.80 ^b^	23.38 ± 0.20 ^c^	18.94 ± ≤ 0.06 ^d^	19.91 ± 0.55 ^d^	
19	Lutein derivative	16.78	nd	19.02 ± ≤ 0.07 ^a^	15.26 ± 0.31 ^b^	8.39 ± 0.18 ^d^		9.70 ± ≤ 0.08 ^c^	7.54 ± ≤ 0.06 ^e^
20	β-Carotene oxygenated derivative	16.97	23.48 ± 0.28	nd	nd	nd	nd	nd	nd
21	Lutein derivative	18.66	14.83 ± 0.11	nd	nd	nd	nd	nd	nd
22	Canthaxanthin	18.81	nd	nd	nd	nd	9.96 ± ≤ 0.07 ^b^	nd	37.30 ± 0.68 ^a^
23	Lutein derivative	19.16	5.35 ± 0.13	nd	nd	nd	nd	nd	nd
24	Unidentified carotenoid	20.70	3.55 ± ≤ 0.03	nd	nd	nd	nd	nd	nd
25	Chlorophyll a derivative	26.73	nd	634.71 ± 3.18 ^e^	1425.40 ± 6.25 ^b^	1796.97 ± 6.44 ^a^	557.64 ± 0.95 ^f^	1050.51± 12.98 ^c^	742.71 ± 17.82 ^d^
26	Echinenone	27.54	nd	48.37 ± 0.45 ^c^	46.27 ± 0.53 ^d^	nd	76.02 ± 0.70 ^a^	9.48 ± 0.17 ^e^	58.25 ± 0.35 ^b^
27	Chlorophyll a derivative	28.17	nd	nd	nd	nd	3588.41 ± 74.03 ^a^	nd	1826.25 ± 57.00 ^b^
28	Chlorophyll-*a*	29.01	1741.99 ± 68.68 ^a^	nd	nd	nd	616.85 ± 4.04 ^b^	nd	456.56 ± 3.29 ^c^
29	Echinenone derivative	29.91	105.81 ± 0.87	nd	nd	nd	nd	nd	nd
30	β-Carotene	35.06		nd	40.76 ± ≤ 0.09 ^a^	15.66 ± 0.21 ^c^	22.96 ± 0.93 ^b^	8.06 ± 0.21 ^d^	nd
31	β-Carotene derivative	37.94	39.46 ± 0.94	nd	nd	nd	nd	nd	nd
Total carotenoids			215.88 ± 4.64 ^c^	383.89 ± 3.54 ^a^	371.43 ± 22.12 ^b^	188.84 ± 0.44 ^d^	162.43 ± 1.29 ^e^	154.33 ±1.68 ^d,e^	118.69 ± 1.07 ^f^
Total chlorophylls			2125.04 ± 65.01 ^c^	634.71 ± 3.18 ^g^	1425.40 ± 6.25 ^e^	1796.97 ± 6.44 ^d^	4762.90 ± 73.72 ^a^	1050.51 ± 12.98 ^f^	3025.52 ± 46.73 ^b^

^1^ Values are expressed as mean ± SD of four determinations. ^2^ nd: Not detected. ^3^ Different superscript letters in the same row denote statistical differences at *p* < 0.05.

**Table 2:** Total phenolic content (TPC) of cyanobacteria extracts ^1,2,3^.

Strains	mg GAE g^−1^
*Phormidium* sp. LEGE 05292	1.52 ± 0.03 ^b^
*Synechocystis salina* LEGE 06099	2.45 ± 0.13 ^a^
*Nodosilinea nodulosa* LEGE 06102	1.23 ± 0,00 ^b,c,d^
*Cyanobium* sp. LEGE 06113	1.41 ± 0.05 ^b,c^
*Synechocystis salina* LEGE 06155	1.18 ± 0.05 ^c,d,e^
*Cyanobium* sp. LEGE 07175	1.09 ± 0.14 ^d,e^
*Tychonema* sp. LEGE 07196	1.07 ± 0.04 ^d,e^

^1^ Expressed in gallic acid equivalents (GAE) g^−1^ of dry biomass. ^2^ Mean ± SD of three independent experiments. ^3^ Different superscript letters denote statistical differences at *p* < 0.05 (ANOVA (analysis of variance), Tukey HSD).

**Table 3:** Inhibitory concentration (IC) values (μg mL^−1^) obtained for the antioxidant activity of cyanobacteria ethanol extracts, for DPPH^•^ and O_2_^•−^^1,2,3^.

Strains	DPPH^•^ (μg mL^−1^)		O_2_^•−^ (μg mL^−^^1^)	
	IC_25_	IC_50_	IC_25_	IC_50_
*Phormidium* sp. LEGE 05292	nd	nd	626.54 ± 0.02 ^a,b^	822.70 ± 0.06 ^b^
*Synechocystis salina* LEGE 06099	481.96 ± 0.09 ^b^	863.82 ± 0.17	nd	nd
*Nodosilinea nodulosa* LEGE 06102	764.14 ± 0.16 ^a,b^	1077.59 ± 0.03	nd	nd
*Cyanobium* sp. LEGE 06113	nd	nd	nd	nd
*Synechocystis salina* LEGE 06155	929.76 ± 0.12 ^a^	nd	756.42 ± 0.74 ^a^	1275.86 ± 0.07 ^a^
*Cyanobium* sp. LEGE 07175	nd	nd	nd	nd
*Tychonema* sp. LEGE 07196	nd	nd	555.54 ± 0.09 ^b^	924.21 ± 0.07 ^b^

^1^ Mean ± SD of three independent experiments. ^2^ nd: Not determined. ^3^ Different superscript letters in the same column denote statistical differences at *p* < 0.05.

**Table 4:** Calibration curves of authentic standards used for quantification of different carotenoids and chlorophylls.

Standards	Calibration Curve	*r* ^2^	LOD (µg/mL) ^a^	LOQ (µg/mL) ^b^
Zeaxanthin	y = 1040515632x − 285181	0.9981	0.0072	0.0239
Lutein	y = 273528935x − 49102	0.9988	0.0921	0.3070
Canthaxanthin	y = 12144399x − 12640	0.9992	0.3251	1.0840
Echinenone	y = 227303816x − 19677	0.9998	0.0935	0.3312
Chlorophyll-*a*	y = 6636898x − 6835	0.9993	0.5143	1.7140
β-Carotene	y = 140882609x − 43144	0.9988	0.0323	0.1080

^a^ LOD: Limit of detection. ^b^ LOQ: Limit of quantification.
